# Integrated mitogenome and Y chromosome analysis untangles the complex origin of African pigs

**DOI:** 10.1016/j.isci.2025.114252

**Published:** 2025-11-27

**Authors:** Lameck A. Odongo, Adeniyi C. Adeola, George M. Msalya, Olawale F. Olaniyan, Ruth N. Njuki, David H. Mauki, Emmanuel K. Ndiema, Xian Shi, Zheng-Fei Cai, Ting-Ting Yin, Yuhua Fu, Xiaolei Liu, Shuhong Zhao, Chabi A.M. S. Djagoun, Pam D. Luka, Ndifor K. Wanzie, George Niba, Olufunke O. Oluwole, Sunday C. Olaogun, Oladipo Omotosho, Oscar J. Sanke, Elliot Greiner, Victor M.O. Okoro, Ofelia G. Omitogun, Philip M. Dawuda, Antoine Souron, Hai-Bing Xie, Bernard Agwanda, Joram M. Mwacharo, Richard P. Bishop, Jian-Lin Han, Min-Sheng Peng, Ya-Ping Zhang

**Affiliations:** 1 State Key Laboratory of Genetic Evolution & Animal Models and Yunnan Key Laboratory of Molecular Biology of Domestic Animals, Kunming Institute of Zoology, Chinese Academy of Sciences, Kunming 650201, China; 2 Sino-Africa Joint Research Center, Chinese Academy of Sciences, Kunming 650201, China; 3 Kunming College of Life Science, University of Chinese Academy of Sciences, Kunming 650204, China; 4 Department of Veterinary Physiology and Biochemistry, Faculty of Veterinary Medicine, Bayero University, Kano, Kano, Nigeria; 5 Sokoine University of Agriculture, Department of Animal, Aquaculture, and Range Sciences, P.O. BOX 3004, Chuo Kikuu, Morogoro, Tanzania; 6 West Africa Livestock Innovation Centre, P.M.B 14, Banjul, The Gambia; 7 School of Agriculture and Environmental Sciences, University of The Gambia, P. O. Box 3530, Serrekunda, The Gambia; 8 Department of Neurosurgery, Institute of Neurological Disease, West China Hospital, Sichuan University, Chengdu 610041, China; 9 National Museums of Kenya, P. O. Box 40658-00100, Nairobi, Kenya; 10 State Key Laboratory for Conservation and Utilization of Bio–Resources in Yunnan, School of Life Sciences, Yunnan University, Kunming 650091, China; 11 Key Laboratory of Agricultural Animal Genetics, Breeding, and Reproduction of the Ministry of Education and Key Laboratory of Swine Genetics and Breeding of Ministry of Agriculture, Huazhong Agricultural University, Wuhan, Hubei, China; 12 Yazhouwan National Laboratory, Sanya, Hainan, China; 13 The Cooperative Innovation Center for Sustainable Pig Production, Wuhan, China; 14 Laboratory of Applied Ecology, Faculty of Agronomic Sciences, University of Abomey–Calavi, Cotonou, Benin; 15 National Veterinary Research Institute, Vom, Nigeria; 16 Department of Zoology, University of Douala, Douala, Cameroon; 17 National Centre for Animal Husbandry, Veterinary and Halieutic Training, Jakiri, Cameroon; 18 Institute of Agricultural Research and Training, Obafemi Awolowo University, Ibadan, Nigeria; 19 Department of Veterinary Medicine, University of Ibadan, Ibadan 200005, Nigeria; 20 Taraba State Ministry of Agriculture and Food Security, Jalingo, Nigeria; 21 The Max Planck Institute for Evolutionary Anthropology, 04103 Leipzig, Germany; 22 Department of Animal Science and Technology, School of Agriculture and Agricultural Technology, Federal University of Technology, Owerri, Nigeria; 23 Department of Animal Science, Obafemi Awolowo University, Ile-Ife, Nigeria; 24 Department of Animal Science, Faculty of Agriculture National University of Lesotho, Roma 180, Lesotho; 25 University of Bordeaux, CNRS, Ministère de la Culture, PACEA, UMR 5199, 33600 Pessac, France; 26 Dryland Livestock Genomics, International Centre for Agricultural Research in the Dry Areass (ICARDA), Addis Ababa, Ethiopia; 27 Animal and Veterinary Sciences, Scotland’s Rural College (SRUC), Roslin Institute Building, Edinburgh, Scotland; 28 International Livestock Research Institute (ILRI), Nairobi, Kenya

**Keywords:** genomics, evolutionary ecology, evolutionary history

## Abstract

The genetic history of African indigenous pigs remains poorly documented due to scarce archaeological and genomic data. Here, we analyzed 473 mitogenomes and 202 Y chromosome sequences from indigenous pigs in Africa, alongside 901 published mitogenomes and 715 Y chromosome sequences from Eurasian pigs and wild boars. Our results reveal that African pigs predominantly descend from European (haplogroup E, 44.8%) and East Asian (haplogroup D, 53.3%) lineages. Interestingly, there was a novel detection of Asian wild boar haplogroup A∗ (1.9%) in Tanzania. This pattern is congruent with that of Y chromosome analysis. Further maternal analyses confirm a genetic link between western African and Iberian pigs dating to about 4.5 ka, and dispersal into eastern Africa coinciding with the Bantu expansion around 2 ka. Our findings demonstrate complex human-mediated dispersal routes, highlighting the role of Bantu societies in shaping the genetic architecture of African indigenous pigs.

## Highlights

• African indigenous pigs show signatures of both European and Asian pigs
• Iberian-type pigs in Africa show a spatial correlation with the Bantu dispersal
• Rare mitochondrial DNA sub-haplogroup A1∗ is identified in pigs from Tanzania

## Graphical abstract

**Figure:**
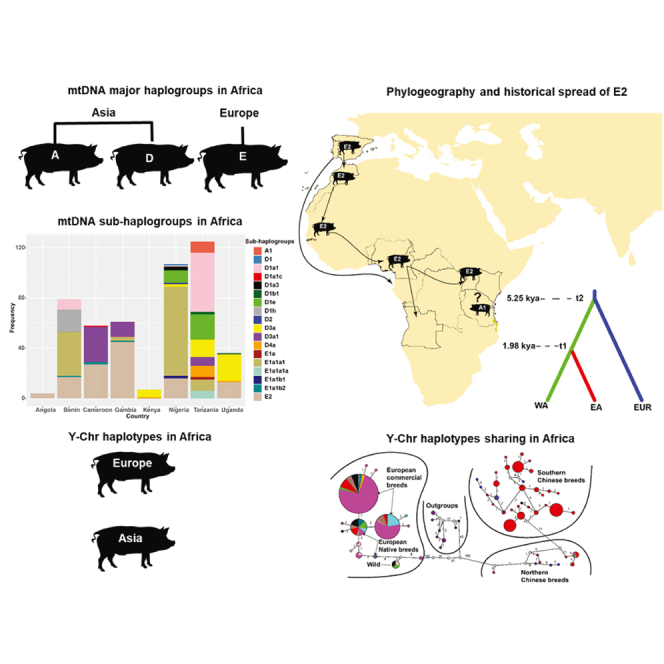

## Introduction

Historically, indigenous pigs (*Sus scrofa domesticus*) were associated with the human populations of the Berber and ancient Egyptians (from Morocco to Egypt), the Sennar (further south along the Nile River up to Sudan), and populations from western-central Africa in Senegambia (Senegal and Gambia), along a west-east band ranging from Nigeria to Sudan (“West African Extension”), and along a north-south band ranging from southern Cameroon to Angola (“Angola Extension”).^1^^,^^2^ Currently, due to cultural reasons, African indigenous pigs are popular with small holder traditional farmers in remote regions in Sub-Saharan Africa and are kept mainly for subsistence.^2^^,^^3^^,^^4^^,^^5^ According to the FAO’s Domestic Animal Diversity Information System, Africa is home to 49 indigenous breeds, with 5% considered endangered and the status of 54% remaining uncertain (http://dad.fao.org). Unlike exotic breeds, African indigenous pigs are tolerant of feed supply irregularities, fibrous and tannin-rich diets, pathogens, and tropical eco-climatic conditions.^6^^,^^7^^,^^8^^,^^9^ They are also highly appreciated by consumers for their marbled meat.^9^ Despite the cultural, economic, and historical importance of African indigenous pigs, their origins remain enigmatic.^1^^,^^10^ While *Sus scrofa* is native to areas of northern Africa,^11^ there is no archaeological or genetic evidence that these populations gave rise to indigenous African pig breeds.^12^ Additionally, the considerable geographic distance (more than 5000 km) between different breeds (such as that between the eastern and western portions of the continent: hereby referred to as western African indigenous pigs and eastern African indigenous pigs) would have likely complicated a single geographic origin scenario for these breeds.^1^^,^^13^^,^^14^

The origins of pig management and husbandry can be traced to the 10th millennium BCE in the Near Eastern and eastern Asia.^15^^,^^16^^,^^17^ Archaeological findings in Egypt revealed pig remains dated to the end of the fifth millennium BCE.^1^ It has been speculated that, during this period, pigs likely spread from the Near East to the Nile Valley basin.^1^ This is partially supported by the finding of Near Eastern mtDNA haplotypes in pig specimens from Egypt’s museum.^10^ Ethnographic data from Sennar in Sudan and Sudan-Ethiopian border indicate that pig farming might have been established in the Nile Valley as early as the Middle Ages, or earlier.^18^ This hypothesis is, however, not supported by archaeological evidence, as early medieval sites like Soba in central Sudan have not yielded any pig remains.^19^ In western-central Africa, pig bones have been discovered at Nkile, Democratic Republic of the Congo, dated to the ninth century.^20^ However, it is still hypothesized that western African indigenous pigs, also known as dwarf pigs (Bakosi, Ashanti, and Elede), originate from Iberian pigs following the introduction of the latter to Africa by Portuguese sailors between the 15th and 19th centuries.^2^^,^^10^ This hypothesis has been supported to an extent by the fact that regions in Africa colonized by the Portuguese adopted lusophone terminologies. For instance, the lusophone word for pig, i.e., “porco,” has been integrated into numerous western Africa dialects.^2^ Linguistic studies have also reported the use of native pig names suggesting earlier pig establishment in Senegambia, and the West African and Angola extension that may be pre-Portuguese.^1^^,^^18^

Over the last 20 years, the two uniparentally inherited genetic marker systems, mitochondrial DNA and Y chromosome, have been widely used to resolve genetic origins, prehistorical range expansions, and demographic processes, in humans, domestic, and wild animals.^21^^,^^22^ Their non-recombinant nature makes it possible for phylogeographic signatures to be inferred as reported for various livestock species.^23^^,^^24^^,^^25^^,^^26^^,^^27^^,^^28^^,^^29^^,^^30^ For instance, Wu et al.^31^ categorized European and Asian pig mtDNA into three major clades: E∗, representing Europe; A∗, Asian wild boar; and D∗, encompassing both eastern Asia wild boars and domestic pigs. These haplogroups were supported by the development of DomeTree (http://dometree.kiz.ac.cn) and MitoToolPy.^32^ Similarly, Ramirez et al.^13^ identified three distinct Y chromosome haplogroups (HY1, HY2, and HY3) in global pig populations. HY1 and HY2 are present in European and eastern Asia domestic pigs and in wild boars, while HY3 is unique to eastern Asia populations. The presence of HY1 and HY2 in eastern Asia pig populations has been postulated to have resulted from sex-biased gene flow between European male and eastern Asia female pigs around 200 years ago.^27^

In African pig populations, genetic analysis revealed the presence of both mtDNA, and Y chromosome markers typically associated with European and Asian pigs.^13^^,^^14^^,^^33^ The Y chromosome markers are categorized into three distinct haplogroups,^13^ highlighting clear genetic clades. However, the mtDNA markers in African pig populations are yet to be standardized into their respective (sub) haplogroups. Nevertheless, phylogeographic analysis of both genetic markers shows that western African pigs share genetic affinity with European pigs, whereas eastern African pigs exhibit genetic similarities to Asian pigs.^13^^,^^14^^,^^33^ But because of the small sample sizes from Africa and the limited resolution of phylogeny based on partial mtDNA cytochrome *b*, D loop sequences, and Y chromosome SNPs of pigs identified in previous studies,^10^^,^^12^ a detailed illustration of the origin and dispersal of domestic pigs in Africa has not yet been developed. Here, we analyzed 1,374 near-complete mitochondrial genomes and 917 sequences of the male-specific Y chromosome (MSY) genes, the DEAD-Box Helicase 3 Y-Linked (DDX3Y), Amelogenin Y-Linked (AMELY), and ubiquitously transcribed tetratricopeptide repeat containing Y-linked (UTY)^13^^,^^34^^,^^35^^,^^36^ to establish a more precise geographic and temporal framework of the evolutionary history and dynamics of modern African indigenous pigs.

## Results

### Phylogeny of African pigs based on mtDNA and MSY sequences

**Figure 1:**
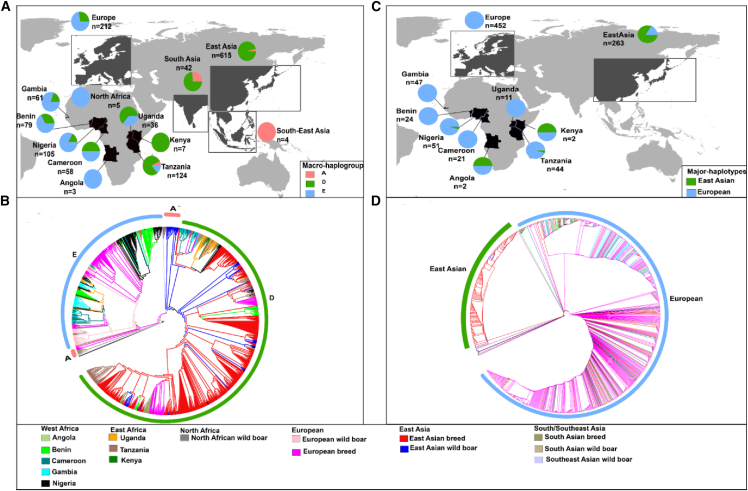
Major haplogroups distribution and phylogenetic positioning of African pigs (A) Map illustrating the geographical distributions of mtDNA haplogroups in African pigs, global pigs, and wild boars. (B) Maximum likelihood tree constructed from 1,352 mitogenomes of African pigs and other global domestic pigs and wild boars. (C) Map displaying geographical distribution of Y chromosome haplotypes of eastern Asian and European pigs in African pigs. (D) Maximum likelihood tree of 917 concatenated Y chromosomal genes (*DDX3Y*, *AMELY*, and *UTY*) sequences of African pigs, eastern Asian, and European domestic pigs and wild boars.

**Figure 2:**
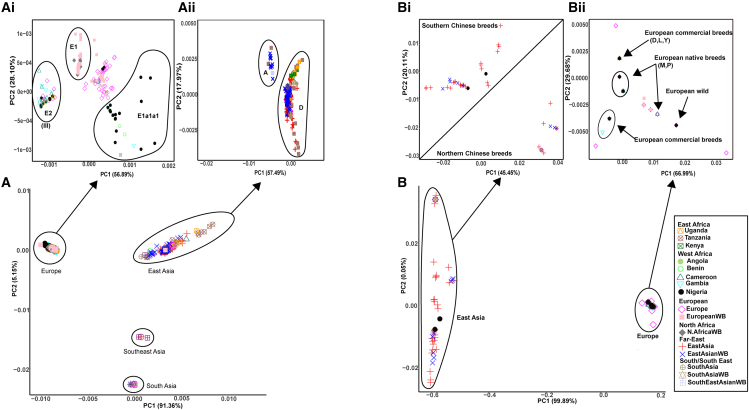
Population structure of African pigs, other global pigs and wild boars inferred from both mitogenome and three concatenated Y chromosome gene sequences (A) Overall PCA plot constructed from the total of 1,352 mitogenomes comprising of African pigs, global pigs, and wild boars. (a.i) PCA plot constructed for mitogenomes projected from the inferred European cluster. (a.ii) Expanded PCA plot of the inferred eastern Asian cluster. (B) Overall PCA plot constructed from 917 concatenated Y chromosome gene sequences. (b.i) PCA plot of concatenated Y chromosome gene sequences extended from eastern Asian cluster. (b.ii) Projected European cluster of concatenated Y chromosome gene sequences.

All three major mtDNA haplogroups A∗, D∗, and E∗, observed in Eurasia, are present in African pigs. Haplogroups E∗ and D∗ are the most abundant, whereas haplogroup A∗ is restricted to eastern Africa, particularly Tanzania, but at a low frequency (Figures 1A, S1, S2, and Data S1A). The maximum likelihood (ML) phylogenetic tree (Figure 1B) nested African pigs within the European and eastern Asian clades, with nine Tanzanian samples representing African haplogroup A∗ positioned at branches just basally to eastern Asian wild boars. For the MSY concatenated gene sequences, the phylogenetic tree (Figures 1C and 1D) also nested African pigs within European and eastern Asian clades. However, the majority (197/202, 98%) of African pig samples are positioned within the European clade, while only about 2% (5/202) are assigned to the eastern Asian clade. The PCA plot for the entire mtDNA dataset (Figure 2A) groups most of the wild boars and domestic pigs according to their geographic origin, except for northern African (Morocco and Tunisia) wild boars, which are grouped with European wild boars. African domestic pigs are grouped within the European and eastern Asian clusters. In the PCA plot of the European cluster (Figure 2ai), local sub-structuring is evident for African pigs, and we designated it as cluster III. The eastern Asian cluster (Figure 2aii) is further divided into two, reflecting the two major clades corresponding to haplogroups A∗ and D∗. Notably, haplogroup A∗ mainly comprises eastern Asian wild boars and nine Tanzanian pigs, whereas haplogroup D∗ includes the remaining African pigs initially assigned to the eastern Asian clade on the ML tree. The PCA results of the entire MSY gene sequences (Figure 2B) reveal a similar structure to the ML tree, with the eastern Asian cluster (Figure 2bi), separating African pigs into those closer to northeastern Asian specimens (i.e., those from Kenya, Uganda, and Angola) and southeastern Asian pigs and wild boar clusters (which included two Nigerian pig samples). The expanded European PCA (Figure 2bii) groups the majority of African pigs with Duroc (D), Yorkshire (Y), and Landrace (L) breeds (referred to as “European commercial”), Mangalitza (M) and Pietrain (P) breeds (referred to as “European natives”), and European wild boars (referred to as “wild”). To further investigate the phylogenetic position of African indigenous pigs in respect to the Near Eastern individuals, we obtained *CytB* sequences of Near Eastern pigs from Ramirez et al.^13^ and analyzed those alongside ours, which were extracted from the mitogenome sequences. The resulting PCA and minimum joining network showed no substantial difference in the clustering pattern revealed by the initial phylogenetic analysis of the mitogenomes for African indigenous pigs (Figures S3 and S4).

### Mitogenomes reveal genetic structure and ancestral lineages of African pigs

**Figure 3:**
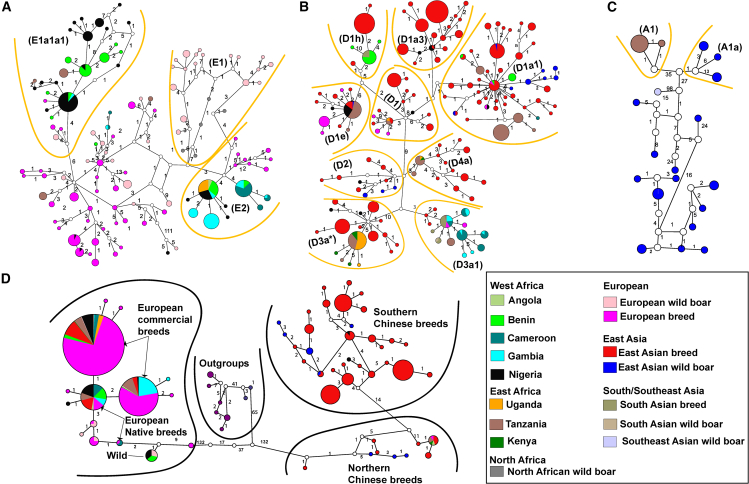
Minimum joining network of African pigs, European and eastern Asian domestic pigs and wild boars inferred from both mitogenomes and Y chromosome gene sequences (A) mtDNA haplotype network constructed from sequences of Haplogroups E. (B) Haplotype network of mtDNA constructed from sequences of Haplogroups D. (C) mtDNA haplotype network representative of Haplogroup A sequences. (D) Y chromosome haplotype network constructed from 917 concatenated Y chromosome gene sequences.

The haplotype networks, shown in Figures 3A and 3C, are organized according to their respective haplogroups (Data S1A). Haplogroup E∗ (Figure 3A) comprises sub-haplogroups E1∗, E1a∗, E1a1a1∗, E1a1a1a∗, E1a1b1∗, E1a1b2∗, and E2∗, which represents African individuals (Figures S1 and S2). The majority of haplotypes fall under sub-haplogroups E1a1a1∗ and E2∗, with E2∗ showing a clear correlation with the local sub-structure denoted as cluster III in the PCA (Figure 2ai). Haplotypes within sub-haplogroup E1a1a1∗ are predominantly found in individuals from Nigeria, Benin, Gambia, and Tanzania, exhibiting close genetic ties to Duroc breed and Italian wild boar haplotypes (Data S1A). On the other hand, sub-haplogroup E2∗ includes haplotypes from individuals in Nigeria, Cameroon, Gambia, Benin, Angola, and Uganda, closely related to Iberian and Yucatan pigs. These haplotypes are exclusive to African pigs (Figure 3A).

Within haplogroup D∗ (Figure 3B), African haplotypes are most frequent in sub-haplogroups D1a1∗, D1e∗, D1h∗, D3a∗, D3a1∗, and D4a∗, while sub-haplogroups D1∗, D1a3∗, and D2∗ show the lowest frequency of African haplotypes. Haplotypes shared between African and non-African pigs are found in sub-haplogroups D1∗, D1a1∗, D1a3∗, D3a1∗, D1e∗, and D1h∗. Notably, a monophyletic group of eastern African pigs is observed within sub-haplogroup D3a∗, with the most common haplotype being shared among pigs from Kenya, Uganda, and Tanzania. This group’s connection to a median vector suggests the absence of unsampled, or potentially extinct haplotypes within African pigs. Tanzanian individuals are particularly prominent within haplogroup D∗, with higher representation compared to other African pigs (as observed in D1∗, D1a1∗, D1e∗, D3a∗, D3a1∗, and D4a∗). For western African pig representatives, sub-haplogroup D3a1∗ is exclusive to Cameroonian and Gambian indigenous pigs, while Benin pigs are found in D1h∗ and D1a1∗. Sub-haplogroup D1e∗ has the highest frequency of Nigerian individuals, whereas D1∗, which also includes haplotypes from Uganda, Tanzania, and Europe, along with D1a3∗, D2∗, and D3a∗, mainly consists of singletons.

In haplogroup A∗ (Figure 3C), haplotypes are exclusive to Tanzanian pigs, predominantly within sub-haplogroup A1∗, which is likely the most ancient within this group. This basal position is congruent with the eastern Asian clade observed in the ML tree (Figure 1D). Sub-haplogroup A1a∗, which includes eastern Asian wild boars, clusters close to A1 despite significant mutational differences. Consistent with the ML tree (Figure 1D) and PCA results (Figures 2B and 2bii), the MSY haplotype network (Figure 3D) groups’ African haplotypes together with European and eastern Asian ones. All three European haplotypes (commercial, native, and wild) are present in African pigs, with the commercial haplotypes occurring at a higher frequency. A few individual African pigs (5/202, 2%) share haplotypes with eastern Asian pigs.

### Divergence time within and between sub-haplogroups E1∗ and E2∗, A1 and A1a

**Figure 4:**
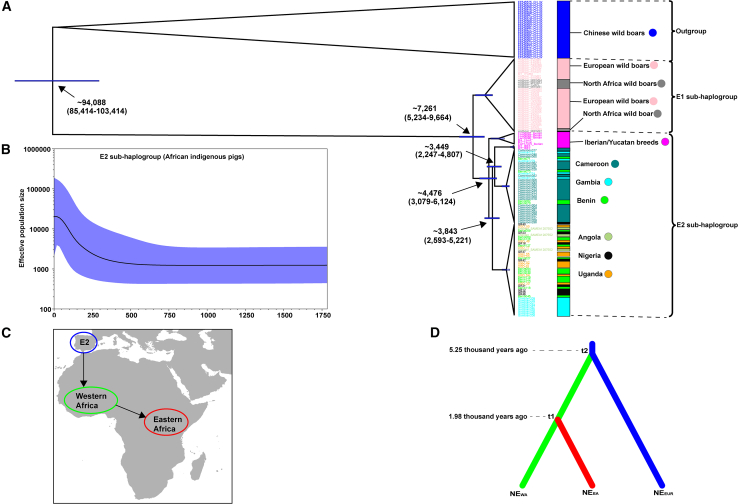
Divergence time, demographic dynamics, and colonization events of African indigenous pigs (A) Divergence time tree showing the split time between African indigenous pigs and Iberian pigs. (B) Bayesian skyline plot depicting effective population dynamics of African indigenous pigs (APs) in sub-haplogroup E2. (C) Map illustrating possible timing and dispersal routes of pigs from Iberian Peninsula into Africa. (D) Colonization routes of the E2 pig sub-haplogroup. The winner scenario is inferred by approximate Bayesian computation statistical approach.

The earliest split was estimated to be approximately 9.7**–**5.2 thousand years (ka) for the divergence of E1∗ (which is exclusive to European and northern African wild boars) and E2∗ (Iberian type pigs) from haplogroup E∗ (Figure 4A), whereas the split between the Iberian pig populations and African indigenous pigs occurred approximately 5.2**–**2.6 ka. The average median divergence time between E2∗ and E1∗ was approximately 7.3 ka and falls within the reported time frame of pig entry into Europe from Anatolia.^37^^,^^38^ By around 5 ka, the European pig genome had lost nearly all traces of the Near Eastern lineage. At this point, 96% of the genetic variants found in European pigs were derived from European wild boar.^24^^,^^39^ African indigenous pigs of sub-haplogroup E2∗ are estimated to have diverged from the Iberian and Yucatan clade around 4.5 ka (Figure 4A). For A1 (Tanzanian pigs) and A1a (Chinese wild boars), the median divergence time occurred at approximately 28 ka.

### Population dynamics of African indigenous pigs

Population dynamics of African pigs were inferred from Bayesian skyline plots (BSP) for sub-haplogroups E2∗ (Figure 4B), E1a1a1∗ (Figure S8), and D3a∗ (Figure S9) mitogenomes. It depicts changes in effective population sizes (*N*_*e*_) over time (Figures 4B, S8, and S9). The E2∗ BSP (Figure 4B) reveals a stable *N*_*e*_ up to around 500 years ago, after which the *N*_*e*_ increases rapidly but seems to stabilize again in recent times. For the E1a1a1∗ African pigs (Figure S8), they exhibited a prolonged stable *N*_*e*_ beginning around 3.5 ka prior to which they had experienced a gradual increase. The stability in *N*_*e*_ persisted until around 500 years ago when a decline commenced subsequently followed by a rapid expansion from around 100 years ago continuing to date. The BSP for D3a∗ pigs show a different pattern to that of E2∗ and E1a1a1∗ (Figure S9). There was a stable *N*_*e*_ up to around 6 ka, followed by a very drastic increase until approximately 4 ka. The population stabilizes for a short period before a decline until around 1 ka from which time it starts to increase once again.

### Dispersal of sub-haplogroup E2∗ pigs into Africa

Using ABC strategy, we constructed the most plausible scenario for the colonization of Africa by sub-haplogroup E2∗ based on 14,979 bps of the mitogenome (Figures 4C and 4D). The scenario with the highest posterior probability (0.5006; 95% CI 0.4908–0.5104) identifies pigs from the Iberian Peninsula as the source population. The founding population for African pigs diverged from their Iberian counterparts around 5.25 ka prior to dispersing into western Africa. Eastern African pigs diverged from western Africa ones around 1.98 ka and dispersed to their current location likely through a terrestrial route (Figures 4C and 4D). Many parameter estimates show high values (>0.2) (Data S2) of relative median of the absolute error (RMAE), which highlights potential concerns regarding the reliability of this scenario, as has been suggested by Kamalakkannan et al.^40^ However, since our primary objective was to investigate the origin of African pig mtDNA subhaplogroup E2 and their dispersal trajectories across Africa, the high RMAE values did not compromise the evaluation of the scenarios we proposed and tested, an observation also acknowledged in the study by Kamalakkannan et al.^40^

Data S2. The ABC prior distributions of the parameters and simulated time estimates of the groups in the colonization models of sub-haplogroup E2

## Discussion

### General diversity and phylogeography of pigs in Africa

Despite the wide variety of local names across the continent, African indigenous pigs are generally considered a single breed with strong ties to Iberian pigs.^10^^,^^41^^,^^42^ These pigs go by several regional names, such as the West African Dwarf pig in Nigeria, Ashanti Dwarf pig in Ghana, “Bush pig” in Togo, Mukota pig in Zimbabwe, Kolbroek in South Africa, Somo in Mali, Olongulu in Angola, and Busia pigs in Kenya.^2^^,^^42^ Our results from both mtDNA and MSY markers of African pigs demonstrate genetic affinities with both European and eastern Asian haplotypes (Figures 1A–1D). Interestingly, this pattern mirrors the phylogeographic structure first described by Ramirez et al.^13^ where most pigs from western Africa cluster with the European clade, while those from eastern Africa align with the east Asian clade in the phylogenetic tree.

### Neolithic human dispersal and introduction of pigs into northwestern Africa

**Figure 5:**
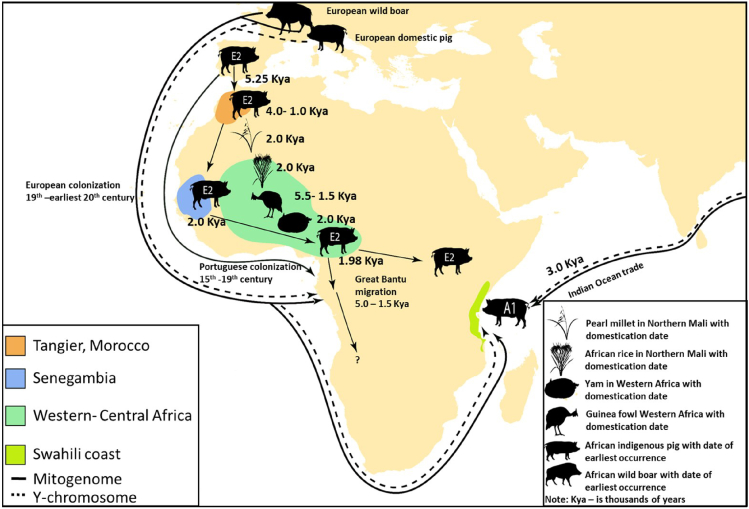
A schematic map illustrates the probable dates of the earliest occurrences of African indigenous pigs from Europe and eastern Asia, as inferred from this study’s mtDNA and Y chromosome analysis The spread of sub-haplogroup E2 (Iberian-type pigs) from western to eastern Africa, aligns with the domestication of African rice,^52^ Guinea fowl,^53^ and the great Bantu dispersal.^54^ In contrast, the presence of sub-haplogroup A1 in eastern Africa, linked to eastern Asian wild boars, indicates earlier connections between eastern Asia and coastal eastern Africa through Indian Ocean trade, see.^55^ The distribution of pigs aligns with ancient African civilizations, supporting the idea that pigs are indicative of a settled farming lifestyle. All silhouettes are sourced from https://www.phylopic.org/.

The genogeographic distribution of mtDNA sub-haplogroup E2∗ pigs, found across the Iberian Peninsula, western Africa, and Uganda (Figures 5, S1, and S2), offers a compelling model for tracing the movement of Iberian pigs (and by extension humans) across Africa. The phylogenetically based origin of the African E2∗ pig clade, which dates back 4.5 ka (Figure 4B), coincides with archaeological evidence of pig domestication by Neolithic populations in Tangier, Morocco.^43^ In line with this, ABC model estimates suggest that Iberian pigs first entered northwestern Africa around 5.25 ka (Figures 4D and Data S2), marking the initial phase of their spread across the continent. The timing of Iberian pigs’ entry into Africa corresponds more closely with the arrival of the Neolithic cultural package in northern Africa and the Iberian Peninsula around 7 to 5 ka.^44^^,^^45^ This connection is further supported by studies from Linstädter et al.,^46^ Zilhão,^47^ and Martínez-Sánchez et al.,^48^ which highlight shared artifacts between northwestern Africa and Iberia Peninsula. Moreover, genetic evidence of introgression between European and African goat populations in Italy and Spain,^49^^,^^50^ further reinforces the long-standing historical genetic exchanges between northwestern Africa and Iberia Peninsula. Pig farming has been an integral part of northern African culture since prehistoric times, particularly among the Berber people, and continued until the advent of Islam.^1^^,^^2^ It is likely that the Berber people introduced pig farming into western sub-Saharan Africa, an idea supported by genetic links between the Fulani people, whose presumed homeland lies in the Gambia and Senegal, and Berber populations from Morocco.^51^ These genetic connections are thought to date back between 1,190 and 670 years ago, with a confidence interval ranging from 2,090 BCE to CE 130.^47^

### Genetic legacy of Bantu expansion and distribution of domestic pigs across Sub-Saharan Africa

The expansion of Bantu-speaking populations stands as one of the most transformative demographic events in late Holocene Africa, significantly reshaping the continent’s linguistic, cultural, and biological landscapes.^54^ This movement, which is estimated to have occurred between 6 and 4 ka, began in western Africa and spread gradually through the Congo rainforest, eventually reaching eastern and southern Africa in a serial-founder fashion.^54^ While our study could not sample individuals from the Congo, the Ugandan pig samples we examined carry the E2∗ sub-haplogroup, which is prevalent in western Africa (Figure S2). ABC model estimates suggest that pigs with the E2∗ sub-haplogroup first dispersed from western Africa into eastern Africa around 1.98 ka (Figure 4D and Data S2). This dispersal coincides spatially—though not necessarily causally—with broader population movements and ecological changes in western and central Africa.^54^ Despite the uncertainty surrounding the exact timing of animal introductions due to the high RMAE value, the broader pattern of Bantu-speaking population dispersals—and the associated movement of domesticated species such as guinea fowl,^53^ pearl millet,^52^ African rice, and yam^56^—may provide a geographic framework for understanding how pigs could have been incorporated into eastward translocations. The presence of pigs in this context is plausible, particularly given historical accounts of feral pigs in regions like Chad during the 19th century.^1^ While documentation of pig introduction during the early period of European contact remains scarce, it is widely accepted that the Portuguese played a key role in this process, particularly along Africa’s coastal regions.^1^ This is reflected in the widespread adoption of the Portuguese term porco for pigs in several local languages.^1^^,^^2^ Therefore, the notable increase in the *Ne* of the collective E2∗ sub-haplogroup in African pigs around 500 years ago in our study (Figure 4B), likely coincides with the onset of Portuguese colonization in Africa during the 16th and 17th centuries AD.^42^ It is plausible that Portuguese colonists encouraged local Bantu farmers to expand pig production, following the population bottleneck of pigs initially caused by the spread of Islam.

### The possible impact of improved British breeds and Indian Ocean trade on African pigs

The high frequency of haplogroup D∗ haplotypes (Figures S1 and S2) suggests that the majority of pigs associated with Tanzania—and more broadly, with coastal eastern Africa—likely originated from a source distinct from those in surrounding inland regions. In this context, the genetic connection between eastern Africa and Asia becomes particularly significant. Several Far-Eastern mitochondrial signatures, such as those embedded in D∗ sub-haplogroups (as defined by MitoToolPy)—including D1a1∗, D1b∗, D1e∗, D1h∗, D3a∗, and D4∗—have been detected not only in eastern African pigs from Uganda and Kenya but also in pig populations across Europe and South Asia.^31^^,^^57^^,^^58^^,^^59^^,^^60^^,^^61^ This shared genetic footprint raises the possibility of at least two plausible routes of introduction into eastern Africa: (i) indirect introgression from improved British breeds brought during the colonial period, which had been extensively hybridized with Chinese sows in the 18th and 19th centuries to select for pigs with earlier reproductive maturity and increased fatness^62^ and (ii) direct introduction to the East African coast with India playing a critical role as a transit point, facilitating the movement of pigs together with other domesticates from eastern and southeastern Asia to Africa, likely during the peak of the Indian Ocean trade.^10^^,^^14^^,^^58^^,^^60^ Supporting the complexity of these historical processes, the high haplotype diversity coupled with low nucleotide diversity observed across all populations (Data S6) provides insight into demographic dynamics. Such a pattern typically suggests population expansion following a bottleneck or a period of low effective population size.^63^ At the same time, this pattern may also reflect the introduction of multiple, genetically distinct populations,^63^ consistent with both colonial and pre-colonial trade-driven introductions. Colonial-era introductions, particularly from Europe, appear to have had a more pronounced influence in western Africa. This is supported by both geographic proximity to Europe and a greater degree of haplogroup sharing with European pigs (Figure 3B). In contrast, coastal eastern Africa seems to have experienced a more complex admixture of sources, with contributions potentially spanning both European colonialism and earlier Indian Ocean trade influences. Further evidence of these dynamics is seen in the substantial increase in *Ne* observed in sub-haplogroup D3a∗, which begins around 4ka (Figure S9) and encompasses all three eastern African pig populations (Uganda, Tanzania, and Kenya). Notably, this timeline aligns with archaeological evidence for the arrival of southeastern Asian domestic chickens at Zanzibar during the late fourth millennium^55^ suggesting contemporaneous movements of other domesticates. Adding to the genetic complexity, we also report the first identification of the rare mitochondrial DNA sub-haplogroup A1∗ in pigs from eastern Africa, specifically in Tanzania (Figures 3C, S1, and S2). The deep divergence time of this haplotype (Figure S17), its basal phylogenetic position (Figure 1B), and its close clustering with Chinese wild boars (Figures S4, S5, and S7) all point to a potential introgression event involving wild pig populations. Given that A1∗ has historically been confined to Asia, its presence in eastern Africa likely reflects gene flow through domesticated pigs that carried introgressed East Asian wild boar genetic material or, it may suggest a direct introduction of East Asian wild boars, which we speculate may have been brought to the region by Portuguese colonists during their presence along the coastal stretches of eastern Africa. However, this remains a hypothesis, as there is currently no direct historical or archaeological evidence to confirm it. We propose this idea as a direction for future research.

Data S6. Genetic diversity indices computed for haplogroup D African indigenous pigs

### The consequences of recent European colonization on African pigs

While we observed the presence of all major European (commercial, native, and wild) and eastern Asian (northern and southern Chinese) MSY haplotypes in the studied African pig populations—consistent with the mtDNA dataset (Figure 3bii and 4D)—European haplotypes were found at a significantly higher frequency than their eastern Asian counterparts. This contrasts with the mtDNA data, where eastern Asian alleles are especially prevalent, particularly in East Africa. One possible explanation for this discrepancy is that eastern Asian MSY haplotypes may have been replaced over time by European ones due to later waves of introduction. For example, Ramirez et al.^13^ reported a high frequency of the Asia-specific HY3 haplotype in Kenyan and Zimbabwean Mukota pigs (35% and 100%, respectively). However, a subsequent study by Noce et al.^14^ found no trace of HY3 in eastern African pigs, supporting the hypothesis that European male lineages have gradually supplanted earlier Asian ones. This pattern aligns with historical records of large-scale European pig imports into Africa, driven by colonial agricultural development and undocumented subsistence-level exchanges.^12^ This pattern is mirrored in our mtDNA results, which show a high frequency of the sub-haplogroup E1a1a1∗ (Figures 3A and S2), linked to the Duroc breed—an exotic European pig developed in Wisconsin from diverse lineages such as Berkshire, Iberian, Tamworth, and Red Guinea Hog.^2^^,^^62^ Crossbreeding between African indigenous pigs and imported European breeds is not uncommon, especially in remote rural areas across the continent.^5^^,^^12^ For instance, in eastern Africa—such as Uganda—the local commercial pig industry has largely transitioned to exotic breeds like Camborough, Landrace, and Large White, often resulting in widespread hybridization.^12^ This trend likely reflects both a recognition of the superior productivity traits associated with exotic breeds and the effects of loose breeding management practices. Accidental interbreeding may be especially common in regions with active or historical restocking programs.^64^ The persistence of mtDNA haplogroup D in African pig populations compared to HY3 may therefore be due to (i) European crossbreeds that trace their maternal lineages to Chinese breeds and (ii) the fact that very few farmers own boars^65^ making pigs carrying HY3 alleles particularly susceptible to genetic bottlenecks. Interestingly, we also detected MSY genetic signatures of European wild boars in several western African pig populations. Historical accounts confirm that European wild boars were introduced to northern, western, and southern Africa during the colonial era, often for use in hunting expeditions,^66^ providing a plausible explanation for their genetic imprint in these regions.

In conclusion, this study sheds light on the genetic diversity and population structure of African indigenous pig breeds, highlighting their importance not only as cultural and economic assets but also as critical reservoirs of genetic resources. Importantly, this research supports the formulation of evidence-based livestock policies in Africa that promote the protection and utilization of indigenous breeds as highlighted by the African Union – Inter-African Bureau of Animal Resources.^12^ By aligning conservation efforts with international and regional agricultural development goals, policymakers can enhance food security, rural livelihoods, and climate resilience across the continent. It is expected that a more detailed depiction of the evolutionary history of African indigenous pigs could be achieved by analyzing high-resolution Y chromosomal markers,^27^ whole-genome variations,^67^ structural variation,^68^ and ancient DNA.^24^^,^^30^ Integrating those molecular data with a thorough revision of the morphology of modern and archaeological specimens would further allow to cross validate timings of divergence and dispersals (e.g.,^38^^,^^69^). Modern morphometric methods have notably proved useful tools to quantify morphological variations in domesticated animals and their closely related wild taxa, and notably in suids (e.g.,^17^^,^^69^^,^^70^). We recommend that future research focuses on identifying and characterizing the mechanisms of adaptive evolution in African indigenous pigs, particularly those conferring resistance to endemic diseases in Africa, to further inform strategies for African indigenous pig conservation, improved survivability, and enhanced meat production.

### Limitations of the study

While our study provides compelling evidence of a genetic connection between Iberian and African pigs, we also recognize certain limitations. Specifically, the small sample size of northern African wild boar mitochondrial DNA data makes it difficult to definitively determine whether the E2∗ haplotypes are derived from native ancient pigs or wild boars. Additionally, it is important to note that while genetic patterns suggest correlations, they do not provide direct evidence of causation, particularly in relation to specific human historical events. For example, in the case of the Bantu migration, the temporal and spatial coincidence of genetic data offers valuable clues but does not establish a clear causal link.

## Resource availability

### Lead contact

Further information and requests for resources and reagents should be directed to and will be fulfilled by the lead contact, Adeniyi C. Adeola (chadeola@mail.kiz.ac.cn).

### Materials availability

This study did not generate new unique reagents.

### Data and code availability

• This paper analyzes existing, publicly available data. The accessions for this data can be found in Data S1A and S1B.
• The Y chromosome variation data reported in this paper (Data S1B) have been deposited in the Genome Variation Map (GVM)^71^ in National Genomics Data Center, Beijing Institute of Genomics, Chinese Academy of Sciences and China National Center for Bioinformation,^72^ under accession number GVM001001. The assembled mitogenomes from this study (Data S1A) have been submitted to GenBank under the accession ID PQ388328-PQ389421.
• No other custom code/software was used for data analysis in the study. The publicly available software and algorithms used in the present study are listed in the key resources table.

## Acknowledgments

We appreciate all the volunteers who contributed to this project. We thank the support from the Animal Branch of the Germplasm Bank of Wild Species; 10.13039/100017417Chinese Academy of Sciences (the Large Research Infrastructure Funding); and West Africa Livestock Innovation Centre, The Gambia. L.A.O is supported by ANSO scholarship for young talent. A.C.A. is supported by the Yunnan Revitalization Talent Support Program: High-end Foreign Expert Project. A.S. is supported by the French government in the framework of the University of Bordeaux’s IdEx “Investments for the Future” program/GPR “Human Past”. This paper contributes to the results framework of CGIAR’s SAPLING Initiative. ICARDA wishes to acknowledge the China government’s contribution to its activities and support from donors to the 10.13039/501100015815CGIAR Trust Fund. This work was supported by grants from the 10.13039/501100021167Sino-Africa Joint Research Center, Chinese Academy of Sciences (SAJC202402 to M.-S.P.); the National Foreign Expert Project (H20240773 to A.C.A.); and the Chinese Academy of Sciences President’s International Fellowship Initiative, Special Expert (2024FSB0002 to A.C.A.).

## Author contributions

A.C.A., M.S.P., Y.P.Z., and J.L.H. conceived and supervised the project; A.C.A., G.M.M., O.F.O., D.H.M., C.A.M.S.D, P.D.L., N.K.W., G.N., J.L.H., R.P.B., O.O.O., S.C.O., O.O., O.J.S., V.M.O.O., and P.M.D. collected the samples; L.A.O., X.S., Z.F.C., R.N.N., and T.T.Y. extracted the genomic DNA and performed the genome sequencing; L.A.O. and X.S. performed variant calling; L.A.O. and Z.F.C. performed population genetic analysis; L.A.O., A.C.A., and M.S.P. drafted the manuscript; H.B.X., E.G., O.F.O., A.S., D.H.M., C.A.M.S.D., P.D.L., E. K. N., O.O.O., S.C.O., O.O., O.J.S., V.M.O.O., P.M.D., O.G.O., X.L., B.A., X.S., Z.F.C., R.N.N., T.T.Y., G.M.M., N.K.W., G.N., J.M.W., J.L.H., Y.F., X.L. S.Z., R.P.B., and Y.P.Z. revised the manuscript.

## Declaration of interests

The authors declare that they have no competing interests.

## STAR★Methods

### Key resources table

**Table:** 

REAGENT or RESOURCES	SOURCE	IDENTIFIER
**Biological samples**		

Whole blood, Ear Muscle	Africa, Asia	N/A

**Critical commercial assays**		

HiSeq 2500 platform	Novogene Bioinformatics Institute	N/A

**Deposited data**		

*S. scrofa* 11.1 reference genome assembly	Warr et al.^73^	https://www.ncbi.nlm.nih.gov/datasets/genome/GCF_000003025.6/
*S. scrofa* mitochondrial reference genome	Lin et al.^74^	GenBank: NC_000845.1
Y chromosome variation data	This paper	https://bigd.big.ac.cn/gvm/getProjectDetail?Project=GVM001001
Assembled mitogenomes	This paper	GenBank: PQ388328-PQ389421

**Software and algorithms**		

fastp(v0.23.0)	Chen et al.^75^	https://github.com/OpenGene/fastp
BWA-MEM (v0.7.17)	Li and Durbin^76^	https://forgemia.inra.fr/gafl/singularity/bwa/-/blob/master/Singularity.bwa_v0.7.17
Samtools (v1.3.1)	Li et al.^77^	https://sourceforge.net/projects/samtools/files/samtools/1.3.1/
GATK (v4.1.4.1)	McKenna et al.^78^	https://github.com/broadinstitute/gatk/releases
Seqtk	N/A	https://github.com/lh3/seqtk/
megahit (v1.2.9)	Li et al.^79^	https://github.com/voutcn/megahit
Aliview (v1.28)	Larsson^80^	https://github.com/AliView/AliView/releases
Plink (v1.9)	Purcell et al.^81^	https://www.cog-genomics.org/plink/
beagle	Browning et al.^82^	https://bio.tools/BEAGLE
vcftools (v0.1.12b)	Danecek et al.^83^	https://sourceforge.net/projects/vcftools/files/vcftools_0.1.12b.tar.gz/
Bcftools	Danecek et al.^84^	https://samtools.github.io/bcftools/bcftools.html
vcf2fasta	N/A	https://github.com/santiagosnchez/vcf2fasta
Online MAFFT	Katoh et al.^85^	https://mafft.cbrc.jp/alignment/server/index.html
MitoToolPy–seq.py	Peng et al.^32^	http://dometree.kiz.ac.cn
ArcMap (v10.7.1)	Docan et al.^86^	https://www.esri.com/zh-cn/home
Tassel (v5)	Bradbury et al.^87^	https://tassel.bitbucket.io/
ggplot2 package in R	R Core Team^88^	https://ggplot2.tidyverse.org/
RaxML (v8)	Stamatakis^89^	https://github.com/stamatak/standard-RAxML
iTOL (v5)	Letunic and Bork^90^	https://itol.embl.de/
FastHaN	Chi et al.^91^	https://github.com/ChenHuaLab/fastHaN
tcsBU	Múrias dos Santos et al.^92^	tcsBU – TCS Beautifier (up.pt)
BEAST (v2.0)	Drummond and Rambaut^93^	https://www.beast2.org/
IQ-TREE web server	Minh et al.^94^	http://iqtree.cibiv.univie.ac.at/
Tracer (v1.7.1)	Rambaut et al.^95^	https://tree.bio.ed.ac.uk/software/tracer/
FigTree (v1.4.4)	N/A	http://tree.bio.ed.ac.uk/software/figtree/
DIYABC (v2.1)	Estoup et al.^96^	http://www1.montpellier.inra.fr/CBGP/diyabc
Arlequin (v3.5)	Excoffier et al.^97^	https://cmpg.unibe.ch/software/arlequin35/Arl35Downloads.html

### Experimental model and study participant details

All animal work was conducted according to a permit (no. IACUCOE202106006) approved by the internal Review Board of Kunming Institute of Zoology, Chinese Academy of Science (CAS), China. Animal sampling in Africa was also approved by local authorities from where the samples were taken.

### Method details

#### Sample information

In this study, we *de novo* assembled a total of 463 near-complete African pig mitogenomes (79 from Benin, 58 from Cameroon, 61 from The Gambia, 105 from Nigeria, 124 from Tanzania, and 36 from Uganda), from whole genome paired-end illumina resequencing reads (Data S1A). The sample collection represented pigs managed under traditional scavenging systems by rural smallholder farmers in sub-Saharan Africa with no known history of crossbreeding with commercial breeds (e.g.,^5^^,^^98^), To minimize relatedness, efforts were made—through farmer questionnaires—to avoid sampling related animals up to the third generation. These were analyzed alongside mitogenomes of domestic pigs and wild boar populations from Europe (*n* = 212), Asia (*n* = 666), northern Africa (*n* = 5) and sub-Saharan African pigs from Angola (*n* = 3) and Kenya (*n* = 7) comprising data that are newly sequenced as well as derived from IAnimal,^99^ GenBank, and Sequence Read Archive (SRA) repository (Figure S10 and Data S1A). We also generated sequences of three MSY genes (*DDX3Y*, *AMELY*, and *UTY*) of male pigs from Uganda (*n* = 11), Tanzania (*n* = 44), Benin (*n* = 24), The Gambia (*n* = 47), Nigeria (*n* = 46), and Cameroon (*n* = 21) and analyzed them together with male pigs data from Europe (*n* = 452), eastern Asia (*n* = 263), additional Nigeria (*n* = 5), Kenya (*n* = 2) and Angola (*n* = 2), comprising individuals derived from IAnimal, SRA and newly sequenced data (Figure S10 and Data S1B and S1C).

### Quantification and statistical analysis

#### Mitogenome assembly

Raw resequencing reads were trimmed of adapters and low-quality bases (<10) using fastp (v0.23.0).^75^ Trimmed reads were retained for assembly if they were ≥150 base pairs (bps) in length with a phred score ≥20, respectively. Reads were then aligned to the *S. scrofa* mitochondrial reference genome^74^ (GenBank number: NC_000845.1) using default parameters with BWA-MEM (v0.7.17).^76^ Sequence Alignment Map (SAM) files were converted to Binary Alignment Map (BAM) files, sorted, indexed, and duplicates identified using samtools (v1.3.1),^77^ picard (http://broadinstitute.github.io/picard/), and GATK (v4.1.4.1).^78^ The list of successfully mapped reads was retrieved by invoking other tools using samtools (v1.3.1),^77^ and consequently used to extract mapped reads from the original fastq files using seqtk (https://github.com/lh3/seqtk/). The extracted mitogenome reads were assembled into contigs using megahit (v1.2.9)^79^ and then aligned against the mitochondrial reference genome using Aliview (v1.28).^80^ The final dataset comprised 1,374 (1,333 newly assembled and 41 from GenBank) mitogenomes spanning a total length of 14,979 bps after excluding gaps and ambiguous bases (Data S1A).

#### Y chromosome variant calling

Using GATK HaplotypeCaller in the GVCF mode and the *S. scrofa* 11.1 reference genome assembly^73^ (https://www.ncbi.nlm.nih.gov/datasets/genome/GCF_000003025.6/), a total of 326,302 high-quality Y chromosomal variants were generated. The detected variants were filtered by the “VariantFiltration” tool with parameters of “QD < 2.0, FS > 60.0, MQ < 40.0, MQRankSum < −8.0”. To minimize false positives in the called variants, the average depth of sex chromosomes and autosomes was calculated individually using samtools (v.1.3.1).^77^ We utilized the differences in depth of coverage of sex chromosomes between female and male individuals to determine the sexes of our African pig samples. We further determined the sexes of all WGS data and validated this using publicly available sample sex information (Figure S11 and Data S1B and S1C). Plink (v1.9)^81^ was used to filter MSY chromosomal variants with the following criteria^100^: (i) removed female individuals and obtained 216,440 Y chromosome SNPs (Figure S12); (ii) retained SNP sites with missing genotype rate of <5%; (iii) removed SNP sites with minor allele frequencies <0.001; and (iv) retained only hemizygous sites in the kept male samples by removing all heterozygous sites (Figure S12). Lastly, similarly to Escouflaire and Capitan,^101^ we used beagle^82^ to impute missing genotypes, and the three MSY genes were extracted and concatenated using vcftools (v0.1.12b) and BCFtools^83^^,^^84^ and then converted into fasta format using vcf2fasta (https://github.com/santiagosnchez/vcf2fasta). The final dataset comprised of 917 male specific concatenated gene sequences with total lengths of 462 SNPs.

#### Haplogroup classification and population structure

The online MAFFT^85^ (https://mafft.cbrc.jp/alignment/server/index.html) was used to perform multiple sequence alignments for the newly assembled and NCBI mitogenomes, and MSY sequences. MitoToolPy–seq.py^32^ was then used to classify mtDNA haplogroups and their geographic distribution was visualized using ArcMap (v10.7.1).^86^ To assess population stratification based on mitogenomes and MSY-chromosome gene sequences, we performed principal component analysis (PCA) using Tassel (v5).^87^ The PCA plot was visualized using ggplot2 package in R.^88^

#### Phylogenetic analysis and network construction

We constructed a maximum likelihood (ML) tree using RaxML (v8)^89^ with the GTRCAT model and 1,000 bootstrap replicates. This model has been found to be efficient and accurate enough with faster computation times for phylogenetic analysis (https://evomics.org/learning/phylogenetics/raxml/). African warthogs (*Phacochoerus africanus*), pygmy hogs *(Porcula salvania)*, and Malaysian bearded pig (*S. barbatus*) were used as outgroups. The ML tree was visualized with iTOL (v5).^90^ To generate the haplotype network, we used FastHaN,^91^ and visualized it using tcsBU^92^ (tcsBU – TCS Beautifier (up.pt)).

#### Genetic diversity, demographic dynamics and divergence time

Haplotype diversity, nucleotide diversity and number of haplotypes for haplogroup D mitogenome sequences were calculated using Arlequin (v3.5).^97^ Arlequin was also used to perform Fu’s *Fs* and Tajima’s *D* neutrality tests to detect evidence of recent population expansion. The significance of the deviations from neutrality were assessed by 1000 coalescent simulations.

To infer demographic dynamics of African specific pig mitogenomes and which ones clustered in sub-haplogroups E2∗, E1a1a1∗ and D3a∗ of the global haplotype networks, we implemented three separate analyses with the Bayesian Skyline plot (BSP) using the random starting tree model in BEAST (v2.0).^93^ The recently published pig mitogenome mutation rate^30^ of 1.2612 x10^−7^ was used to calibrate the BSP. We performed each run three times starting from a random tree with a Markov Chain Monte Carlo (MCMC) simulation for 60 million generations, sampled every 6,000 generations with the first 10% generations discarded as burn-in. For this analysis, we invoked the HKY substitution model without site invariants, which was determined using IQ-TREE web server^94^ (http://iqtree.cibiv.univie.ac.at/) to be the best fit model. A strict molecular clock model was applied, given its suitability for phylogenies with shallow roots due to low rate variation among branches.^102^^,^^103^ Additionally, we used the coalescent Bayesian Skyline tree prior with 10 groups under a piecewise-constant skyline model to capture population size changes over time. Tracer (v1.7.1)^95^ was used to assess convergence across runs.

Bayesian divergence tree between the mitogenome sub-haplogroups E1∗ and E2∗ was generated using BEAST (v2.0). We employed a random starting tree model with a constant population size coalescent tree prior, incorporating eastern Asian wild boars (*S. scrofa*) from Wu et al.^31^ as the outgroup. The mutation rate of 1.2612 x 10^−7^ was used to calibrate the divergence times. The analysis was conducted by employing an MCMC chain length of 10 million generations, sampling trees every 10,000 generations with a strict clock and using the HKY substitution model without site invariants. We also calculated the divergence time between the mitogenome sub-haplogroup A1 and A1a. We tested both a strict clock and an uncorrelated lognormal-distributed relaxed clock under HKY+G+I. For both models three MCMC runs with 30,000,000 iterations were run, with 10,000 sampling frequency. The first 10% of the generations were discarded as burn-in. The estimated effective sample size (ESS) for all parameters was greater than 200, as determined using Tracer (v1.7.1). The two models were compared with a marginal likelihood estimation using general stepping-stone sampling (GSS) (https://beast.community/model_selection_2). The strict clock provided higher effective sample size (ESS) values because of earlier chain convergence; therefore, it was the chosen model to generate divergence tree. Tree visualization was performed using FigTree (v1.4.4) (http://tree.bio.ed.ac.uk/software/figtree/).

#### Inference of mtDNA sub-haplogroup E2 colonization history in Africa

To investigate possible colonization trajectories of the sub-haplogroup E2∗ in Africa, we used the Approximate Bayesian Computation (ABC) to test multiple scenarios of dispersal using the near complete (14,979 bps) mitogenome sequences. Simulations were performed using DIYABC (v2.1).^96^ We constructed the dispersal scenarios based on the ML phylogenetic and divergence time tree results as well as archaeological and genetic inferences of European pig domestication from wild boars discerned from previous studies.^37^^,^^104^ We divided lineage E2∗ into three metapopulations according to geographic distributions: (i) European-Iberian Peninsula group (EUR) comprising Iberian and Yucatan pigs; (ii) western Africa (WA) group comprising pigs from Angola, Benin, Cameroon, Gambia, and Nigeria; and (iii) eastern Africa (EA) group which included only Ugandan pigs within the sub-haplogroup E2∗. To test our hypothesis of an African origin of E2∗ lineage and determine its dispersal routes across the continent, we implemented an independent model invoking two scenarios that considered the two African pig groups (WA and EA) as the ones contributing to the main difference in the sub-haplogroup (Figures 4D and S13).

Starting with EUR (NE_EUR_) as the source population: (i) the first dispersal was postulated to be from EUR to WA (NE_WA_) at time t2 and posteriorly from WA to EA (NE_EA_) at time t1 (Figure 4D), and (ii) the expansion begun from EUR to EA at time t2 and posteriorly from EA to WA at time t1 (Figure S11). The HKY substitution model was used as selected using IQ–TREE web server to be the best fit. The mean mutation rate was set as 10^−8^ to 10^−7^ per site per generation.^105^ The statistical summaries (SS) were selected after PCA analysis (Figure S14) to pre-evaluate the similarity between the simulated and empirical datasets through the “evaluate scenario-prior combination option” which checks whether the models together with the chosen prior distributions have the potential to generate a subset of summary statistics close to the observed summary statistics.^105^ The SS for the simulated dataset, which were considered under the one-sample SS, included mean of pairwise differences, mean of number of rarest nucleotides at segregating sites, and variance of numbers of the rarest nucleotides at segregating sites. The two-sample SS included number of haplotypes, number of segregating sites, mean of pairwise differences and *F*_*ST*_. After simulating one million datasets for each of the two scenarios and used both a direct and logistic regression method to compare the posterior probability of scenarios based on the selected summary statistics. The precision of each parameter estimates was evaluated by computing the relative median of the absolute error (RMAE).^105^ Finally, the model was verified by calculating the goodness-of-fit statistics of the winner scenario from the observed dataset and visualized using PCA (Figures S15 and S16).

## Supplemental information

Document S1. Figures S1–S17

Data S1. Information for datasets and samples used in this study (A) Mitogenomes used, their inferred haplogroups and population IDs. (B) Y chromosomes used based on the validity of male sexes of the studied populations. (C) Depth coverage values of X, Y, and autosomes with the inclusion of both X/autosome and Y/autosome ratios used to validate the Y chromosomes.

Data S3. Reported presence of earliest occurrences of pigs in Africa

Data S4. General stepping-stone sampling used for marginal likelihood estimates of strict and relaxed clock

Data S5. Eigenvalues used to compute variance percentages of mitogenome and Y chromosome sequences
