# Supplementary material for: Integrated mitogenome and Y chromosome analysis untangles the complex origin of African pigs
Source: iScience. 2025 Nov 27;29(1):114252. doi: 10.1016/j.isci.2025.114252 (PMC12775879; doi:10.1016/j.isci.2025.114252)
Supplement: Document S1. Figures S1–S17 [file mmc1.pdf]

iScience, Volume *29*

Supplemental information

Integrated mitogenome and Y chromosome analysis untangles the complex origin of African pigs

Lameck A. Odongo, Adeniyi C. Adeola, George M. Msalya, Olawale F. Olaniyan, Ruth N. Njuki, David H. Mauki, Emmanuel K. Ndiema, Xian Shi, Zheng-Fei Cai, Ting-Ting Yin, Yuhua Fu, Xiaolei Liu, Shuhong Zhao, Chabi A.M. S. Djagoun, Pam D. Luka, Ndifor K. Wanzie, George Niba, Olufunke O. Oluwole, Sunday C. Olaogun, Oladipo Omotosho, Oscar J. Sanke, Elliot Greiner, Victor M.O. Okoro, Ofelia G. Omitogun, Philip M. Dawuda, Antoine Souron, Hai-Bing Xie, Bernard Agwanda, Joram M. Mwacharo, Richard P. Bishop, Jian-Lin Han, Min-Sheng Peng, and Ya-Ping Zhang

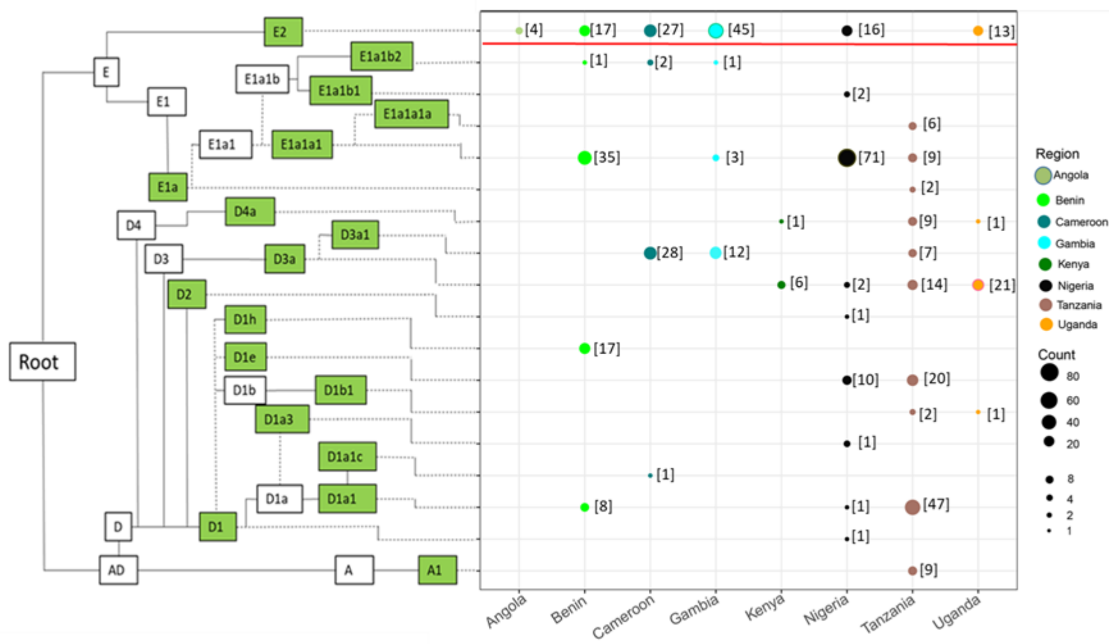

Figure S1: Distribution of mtDNA haplogroups in the African pigs. Pig populations are highlighted with different colors as size of the circle and numbers in the bracket represent numbers of individuals.

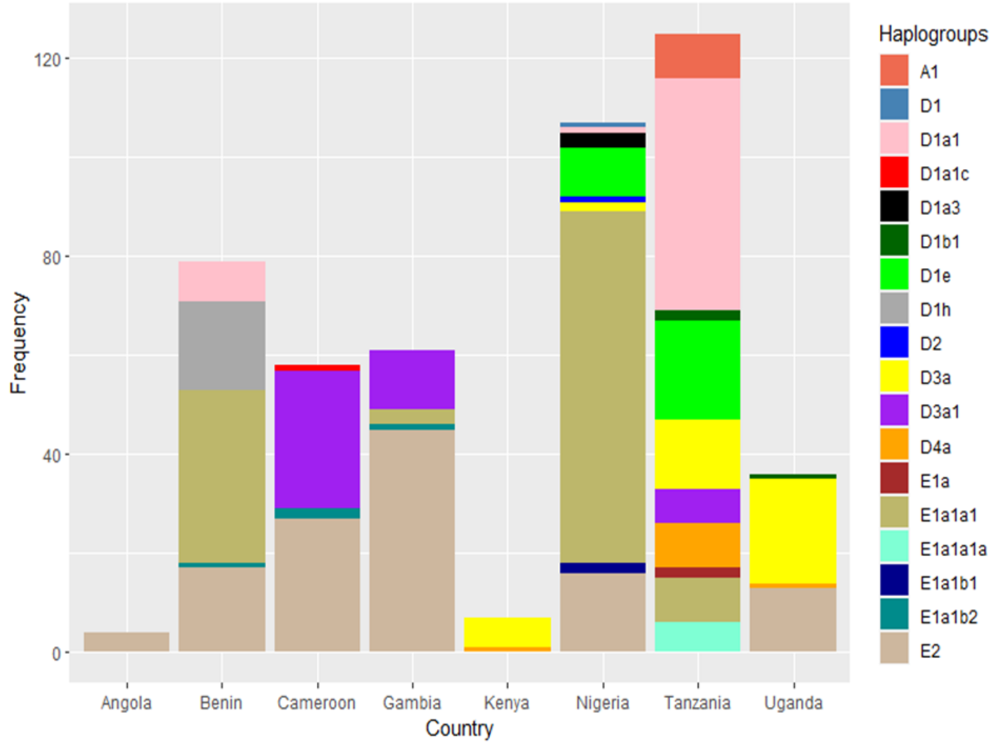

Figure S2: Bar plot of mtDNA subhaplogroups in the African pigs. The colors represent different sub haplogroups of pigs observed in Africa.

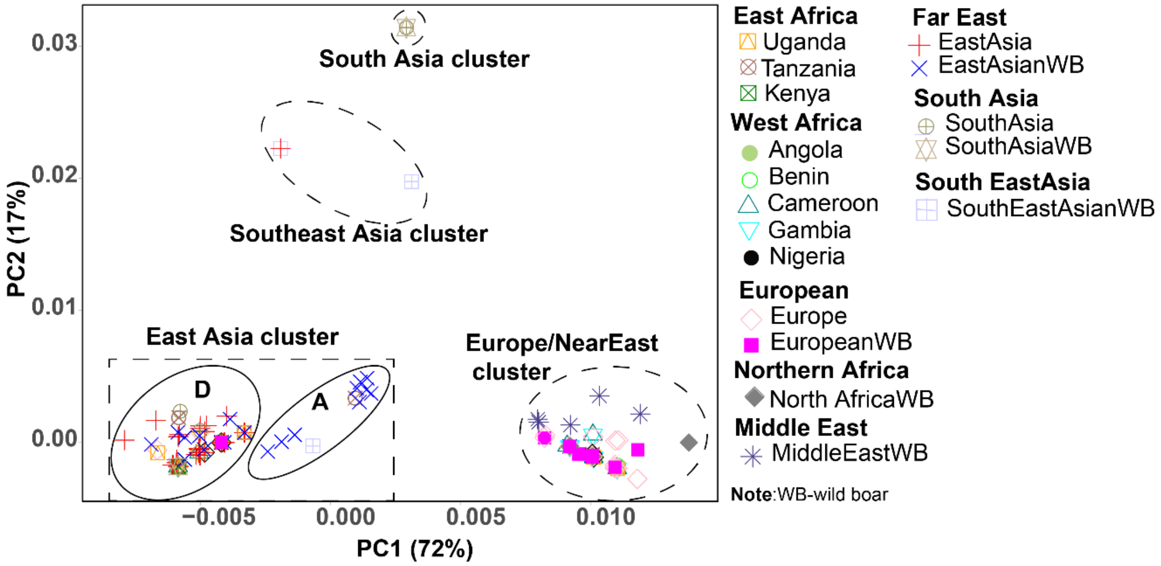

Figure S3: PCA analysis inferred from *CYTB* sequences of Europe, Asia, Northern and Sub-Saharan African pig and wild boar populations, together with the Near Eastern (Middle East) sequences retrieved from Ramirez et al. (2009).

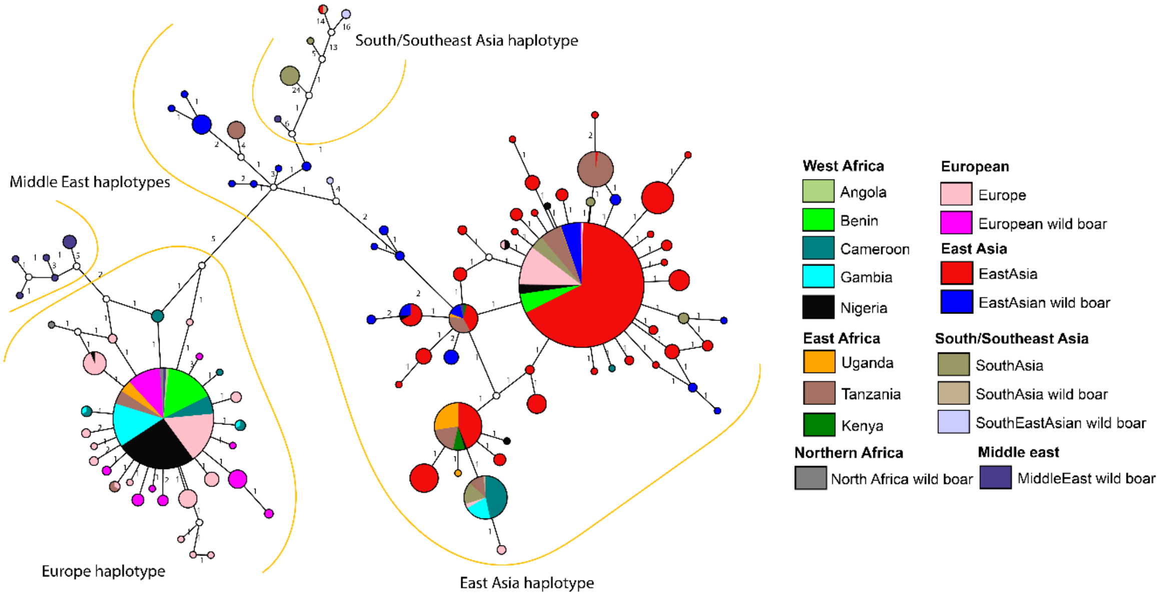

Figure S4: Minimum joining network analysis inferred from *CYTB* of Europe, Asia, Northern and Sub-Saharan African pig and wild boar populations, together with the Near Eastern (Middle East) sequences retrieved from Ramirez et al. (2009).

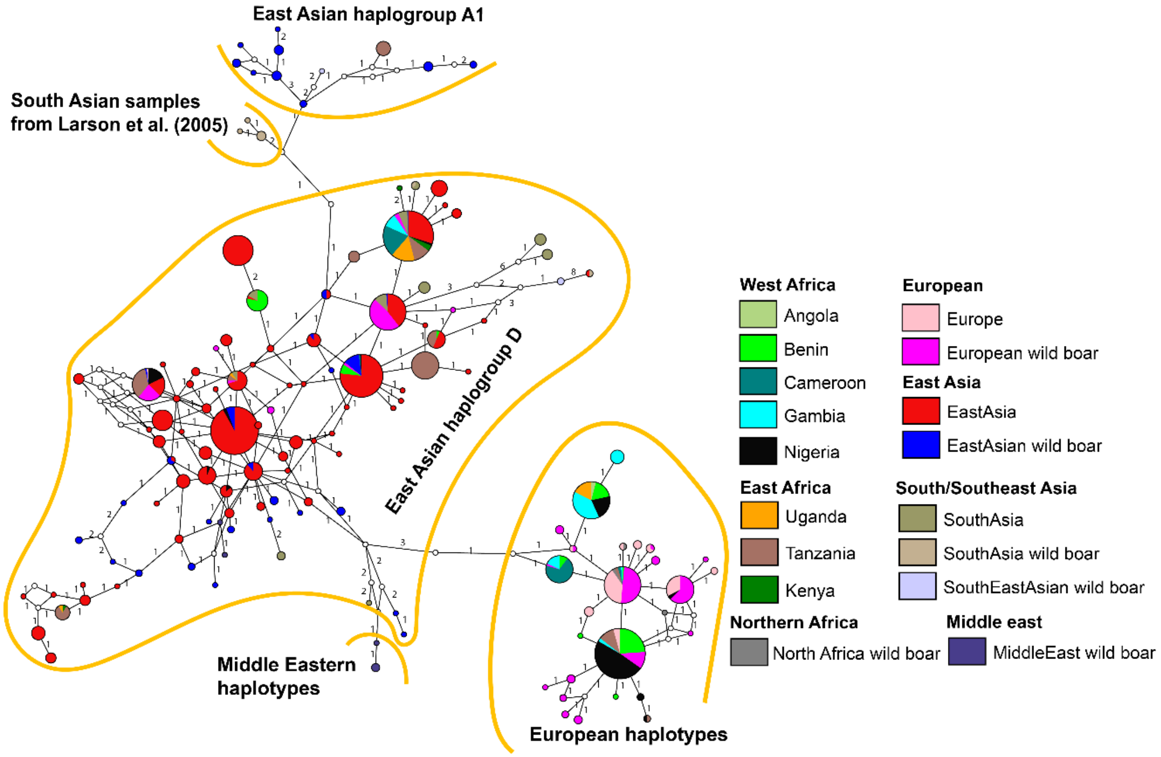

Figure S5: Minimum joining network analysis inferred from mtDNA control region (D-loop fragment) of Europe, Asia, Northern and Sub-Saharan African pig and wild boar populations, together with the South Asian wild boar sequences (specifically from India) retrieved from Larson et al. (2005) that could not be assigned to haplogroup A1 or E as reported by Wu et al.(2009).

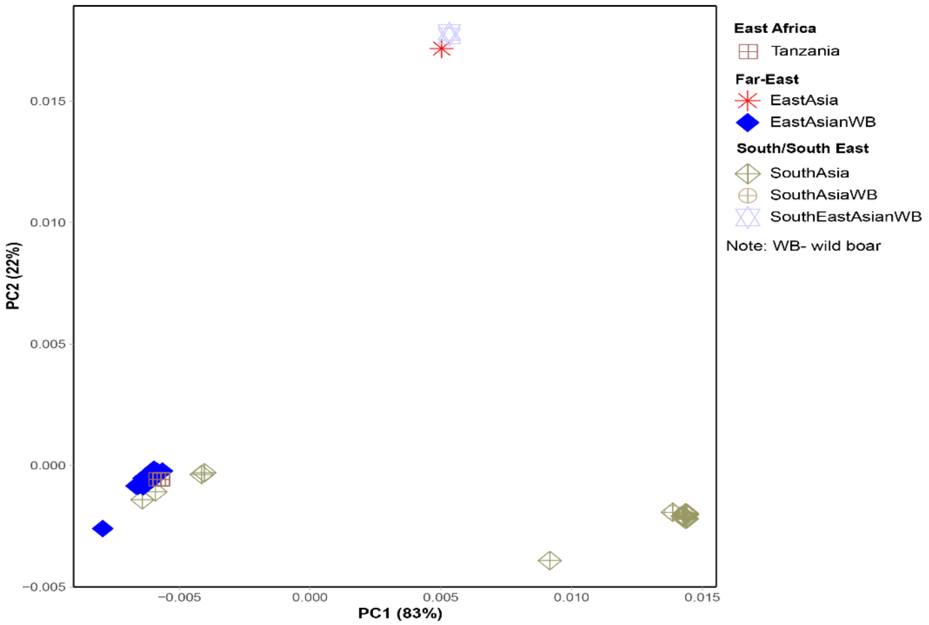

Figure S6: PCA analysis inferred from all haplogroup A* sequences of East Asia, South Asia, Southeast Asia and Tanzanian pigs and wild boar populations.

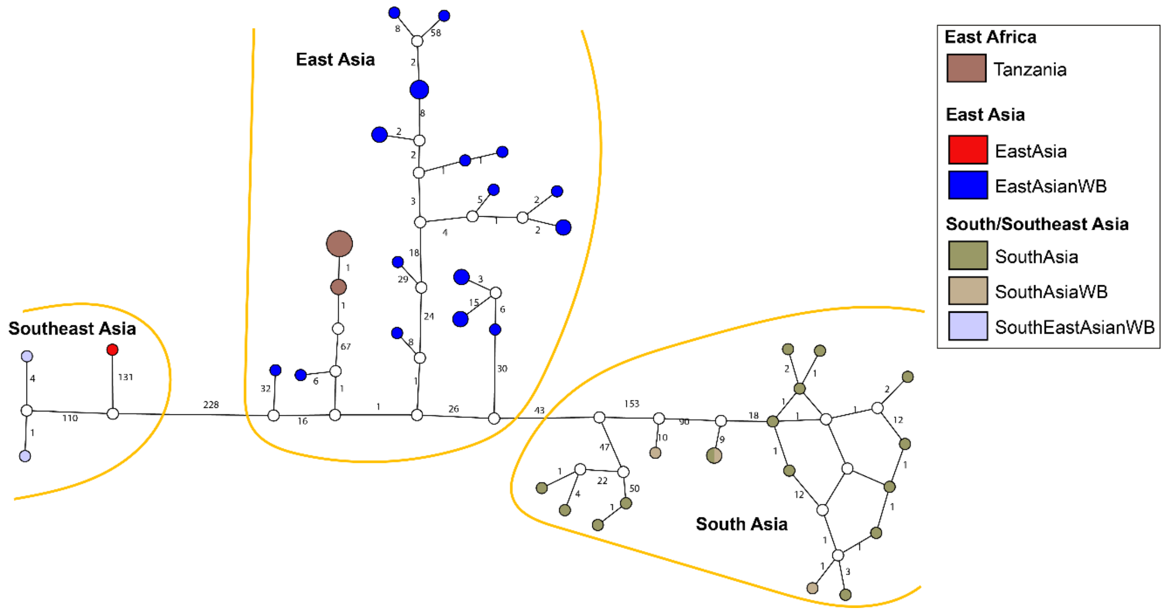

Figure S7: Minimum joining network analysis inferred from all haplogroup A* sequences of East Asia, South Asia, Southeast Asia and Tanzanian pigs and wild boar populations

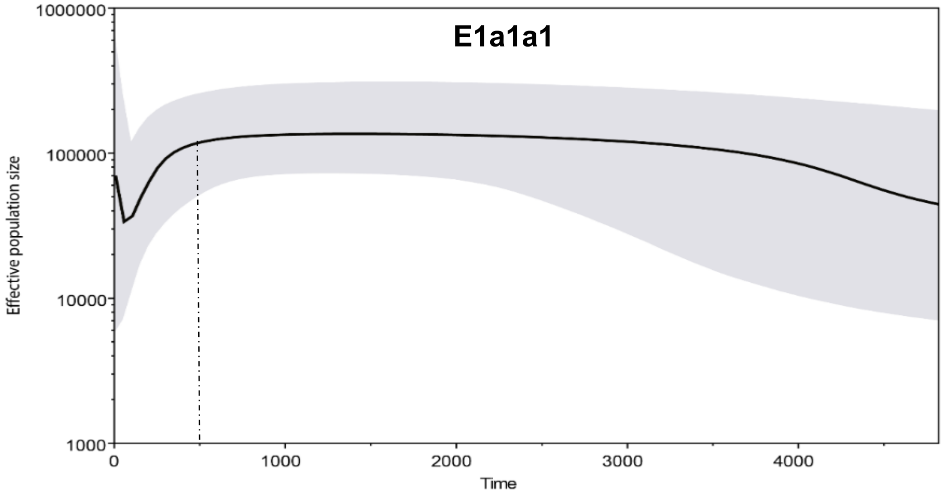

Figure S8: Near complete mitogenomes inferred Bayesian skyline plot of Sub haplogroup E1a1a1 of African indigenous pigs. The 95% highest posterior density (HPD) is highlighted by a gray-shaded area. The X-axes represent time in thousands of years before present, while the Y-axes represent the mean effective population sizes.

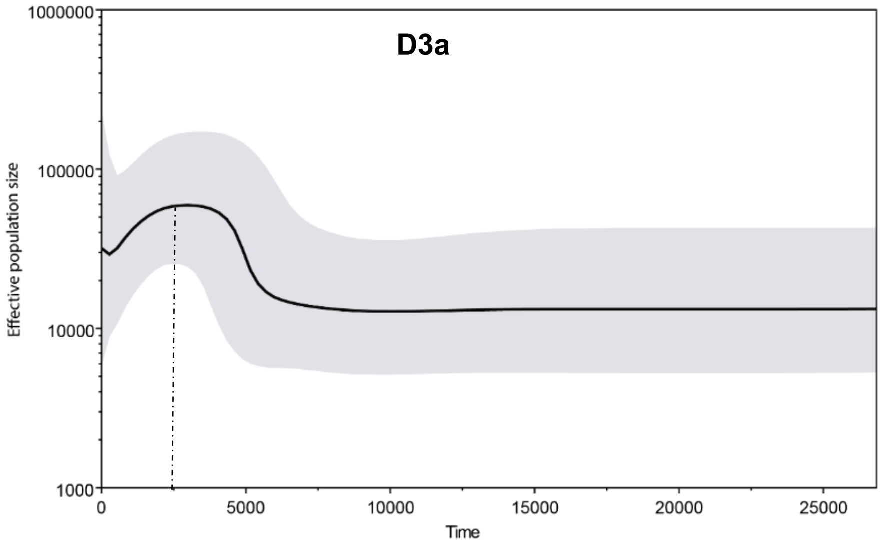

Figure S9: Near complete mitogenomes inferred Bayesian skyline plot of Sub haplogroup D3a of African indigenous pigs. The 95% highest posterior density (HPD) is highlighted by a gray-shaded area. The X-axes represent time in thousands of years before present, while the Y-axes represent the mean effective population sizes.

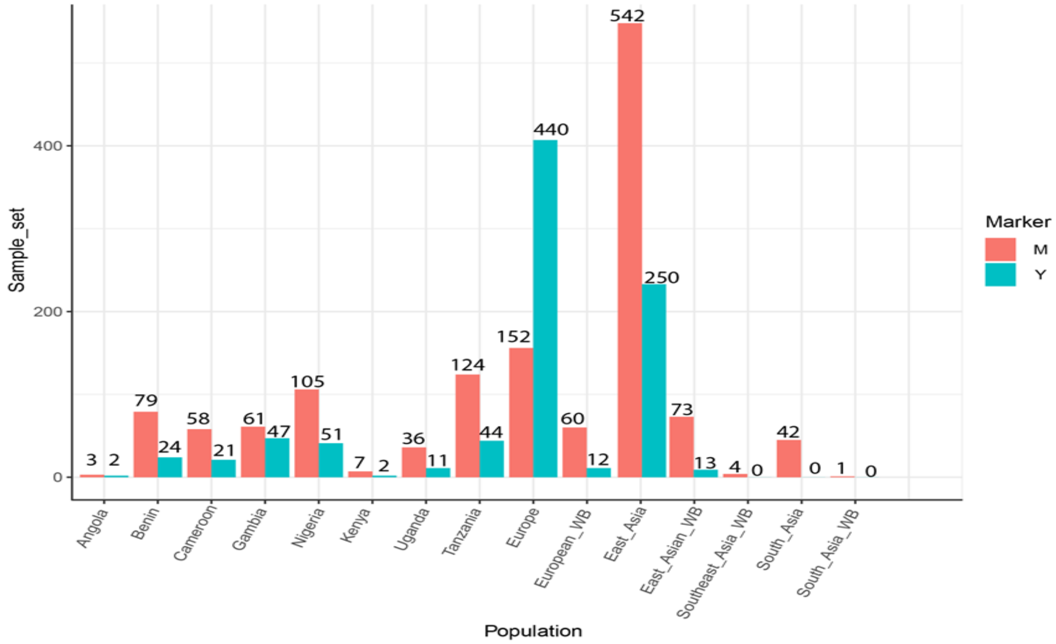

Figure S10: Bar plots of generated datasets. The colors represent the two-uniparental markers. The letters M is mitogenome and Y is the Y chromosome sequences generated from Whole genome sequencing data.

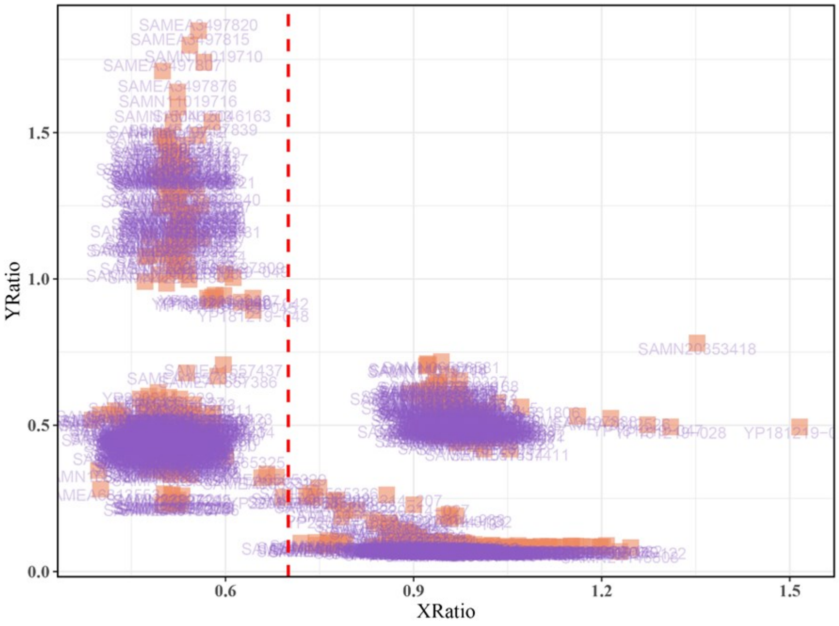

Figure S11: Scatterplot of Y/autosomes and X/autosome coverage depth ratio. The Y-axes is indicative of the male specific samples and the X-axes is indicative of the female samples.

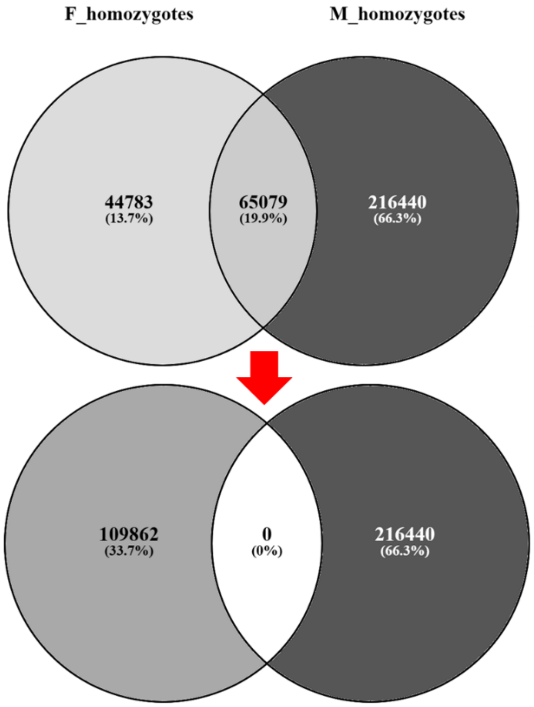

Supplementary Fig S12: Venn diagram of homozygous SNPs in the inferred Females (F) and Males (M). The overlapping homozygous SNPs were removed from the inferred Males samples to ensure we retained only male specific homozygous SNPs.

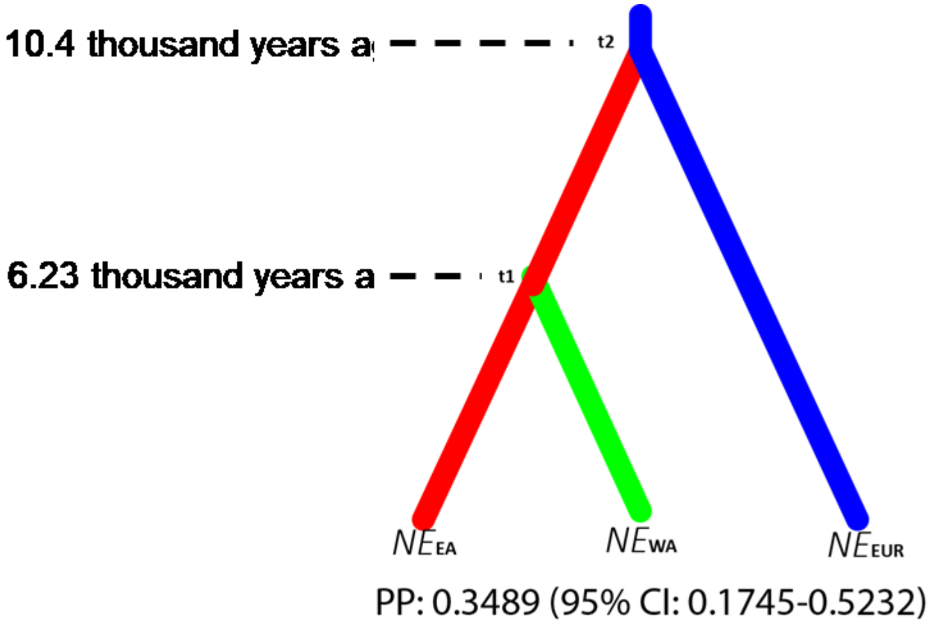

Figure S13: Alternate candidate model for population recolonization of domestic pig of sub-haplogroup E2 in Africa. This is scenario 2, which represents dispersal from eastern Africa to western Africa from Iberian Peninsula.

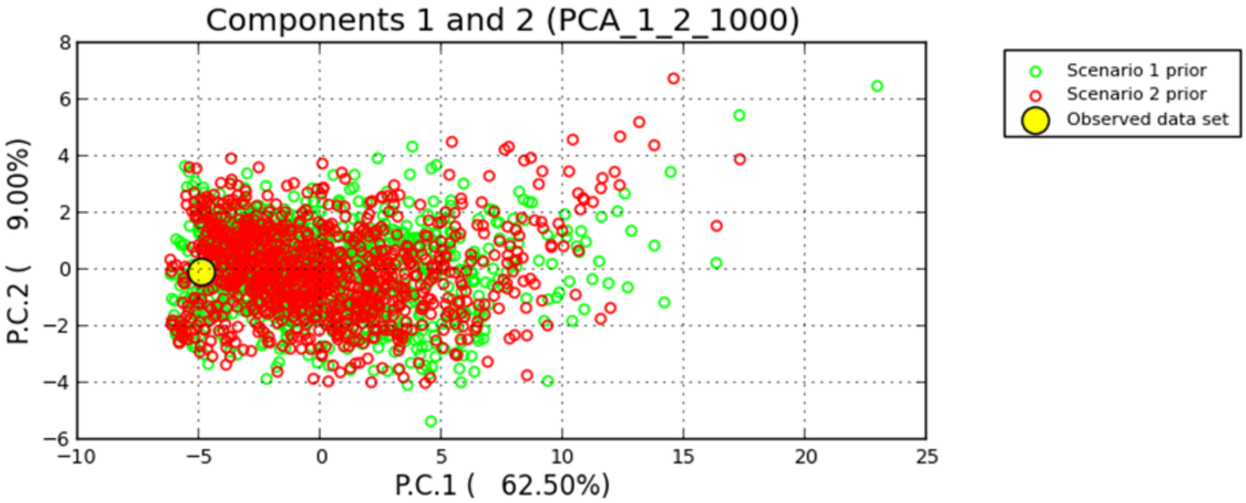

Figure S14: PCA analysis to pre-evaluate the similarity between the simulated and empirical datasets between scenario 1 and 2 based on comparison of prior distribution of parameters. Scenario 1 represents dispersal from Iberian Peninsula to western Africa and finally to eastern Africa and scenario two represents dispersal from eastern Africa to western Africa from Iberian Peninsula.

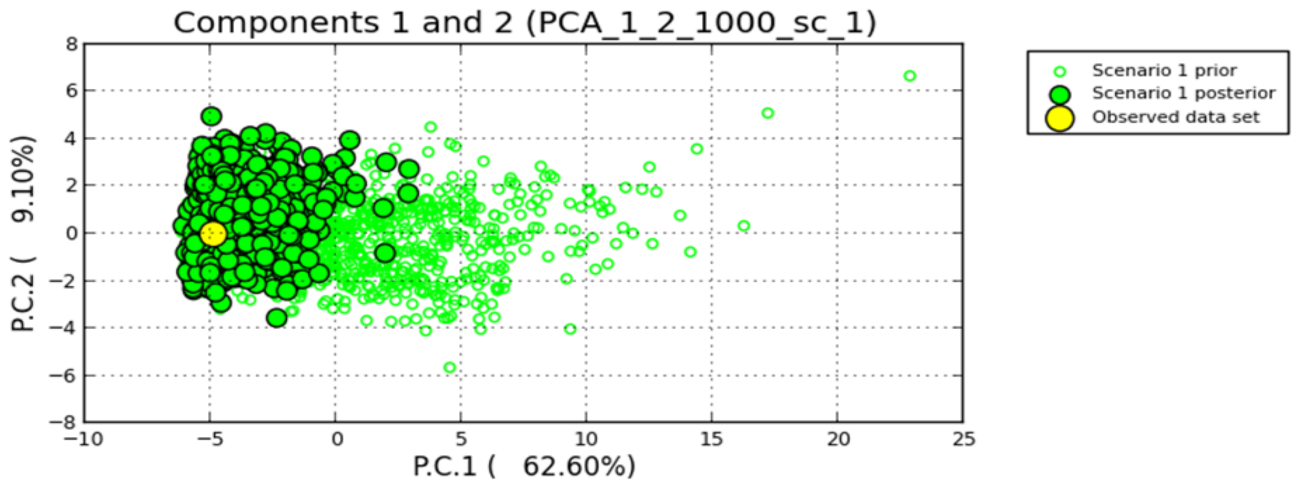

Figure S15: PCA analysis to evaluate the similarity between the simulated and empirical datasets between scenario 1 prior and posterior distribution of parameters.

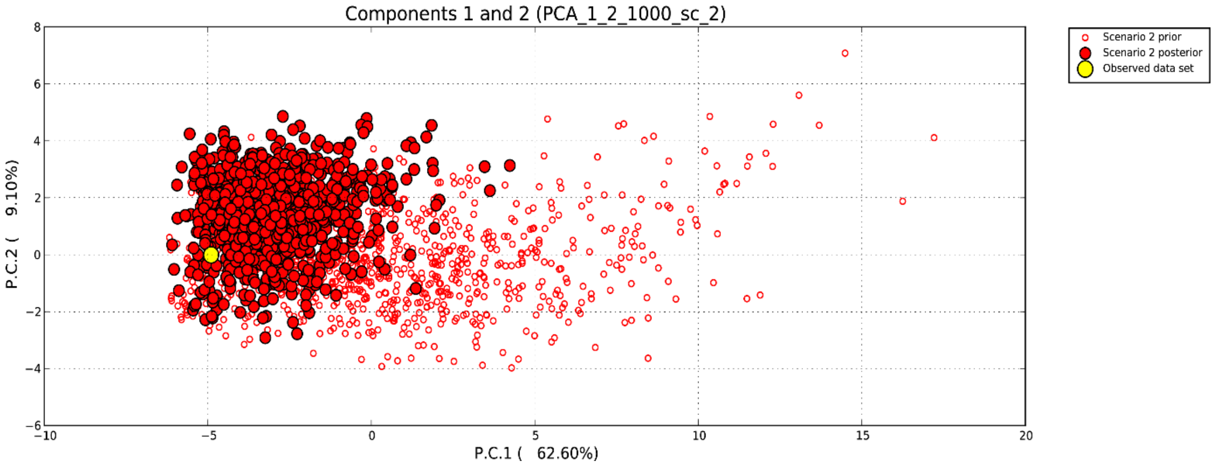

Figure S16: PCA analysis to evaluate the similarity between the simulated and empirical datasets between scenario 2 prior and posterior distribution of parameters.

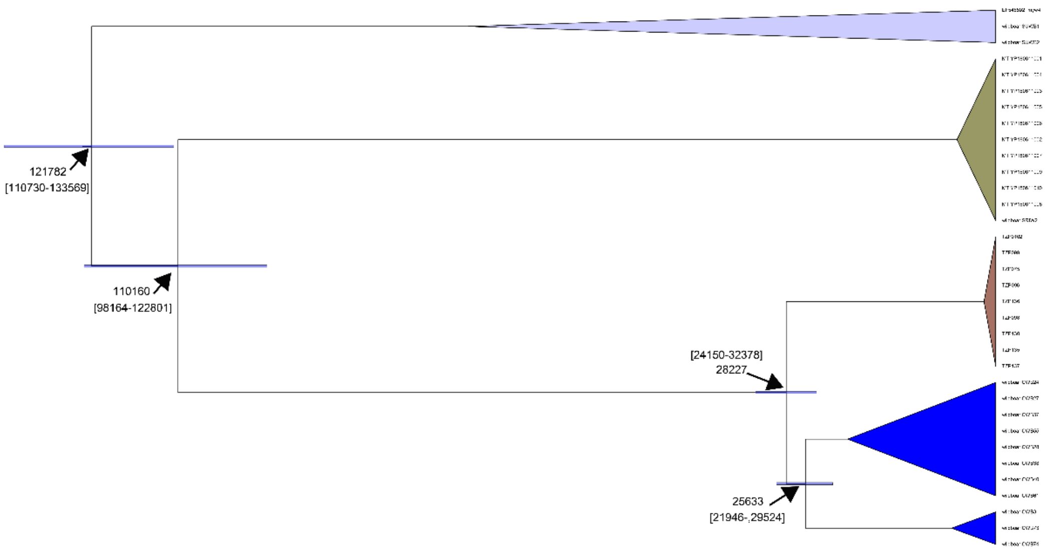

Figure S17: Divergence time tree between Tanzanian A1 and Chinese wild boars of A1a mtDNA subhaplogroups.
